# Mycovirus Fusarium oxysporum f. sp. dianthi Virus 1 Decreases the Colonizing Efficiency of Its Fungal Host

**DOI:** 10.3389/fcimb.2019.00051

**Published:** 2019-03-12

**Authors:** Almudena Torres-Trenas, Pilar Prieto, M. Carmen Cañizares, María Dolores García-Pedrajas, Encarnación Pérez-Artés

**Affiliations:** ^1^Departamento de Protección de Cultivos, Instituto de Agricultura Sostenible, Consejo Superior de Investigaciones Cientificas, Córdoba, Spain; ^2^Instituto de Hortofruticultura Subtropical y Mediterránea “La Mayora”, Universidad de Málaga, Consejo Superior de Investigaciones Científicas, Málaga, Spain; ^3^Departamento de Mejora Genética, Instituto de Agricultura Sostenible, Consejo Superior de Investigaciones Científicas, Córdoba, Spain

**Keywords:** *Fusarium oxysporum*, carnation, chrysovirus FodV1, hypovirulence, CLSM microscopy, mycovirus-dissemination

## Abstract

Mycoviruses that induce hypovirulence in phytopathogenic fungi are interesting because their potential use as biological control agents of the plant diseases caused by their fungal hosts. The recently identified chrysovirus Fusarium oxysporum f. sp. dianthi virus 1 (FodV1) has been associated to the induction of hypovirulence in *Fusarium oxysporum* f. sp. dianthi, the forma specialis of *F. oxysporum* that causes vascular wilt in carnation (*Dianthus caryophyllus*). In this work, we have used confocal laser scanner microscopy and two isogenic GFP-labeled strains of *F. oxysporum* f. sp. *dianthi* infected (V^+^) and not infected (V^−^) with the Fusarium oxysporum f. sp. dianthi virus 1, respectively, to analyze the effect of mycovirus FodV1 on the plant colonization pattern of its fungal host. Results demonstrate that FodV1-viral infection affects the speed and spatial distribution of fungal colonization into the plant. Initial stages of external root colonization were similar for both strains, but the virus-free strain colonized the internal plant tissues faster than the virus-infected strain. In addition, other differences related to the specific zone colonized and the density of colonization were observed between both *F. oxysporum* f. sp. *dianthi* strains. The hyphae of both V^−^ and V^+^ strains progressed up through the xylem vessels but differences in the number of vessels colonized and of hyphae inside them were found. Moreover, as colonization progressed, V^−^ and V^+^ hyphae propagated horizontally reaching the central medulla but, while the virus-free strain V^−^ densely colonized the interior of the medulla cells, the virus-infected strain V^+^ appeared mainly in the intercellular spaces and with a lower density of colonization. Finally, the incidence of FodV1-viral infections in a collection of 221 isolates sampled between 2008 and 2012 in the geographic area where the originally infected isolate was obtained has been also analyzed. The very low (<2%) incidence of viral infections is discussed here. To the best of our knowledge, this work provides the first microscopic evidence about the effect of a hypovirulence-inducing mycovirus on the pattern of plant colonization by its fungal host.

## Introduction

Fungal viruses (mycoviruses) are found infecting the main taxonomic groups of fungi, including phytopathogenic species. Although there are a few recent evidences that extracellular transmission may occur (Yu et al., 2013; Liu et al., 2016; Marzano et al., 2016), mycoviruses are mainly transferred between strains by hyphal anastomosis (horizontal transmission), and during the production of spores (vertical transmission). Most of the viral infections in fungi are cryptic, i.e., no appreciable changes are observed in the phenotype of the host, but in some cases an alteration of particular phenotypic traits occurs as a consequence of the viral infection. Mycoviruses inducing hypovirulence in phytopathogenic species, i.e., that reduce the virulence of the fungus against its host plant, are the most interesting to study, because the possibility of using them as biological control agents of the diseases caused by their fungal hosts (reviewed in: Ghabrial and Suzuki, 2009; Pearson et al., 2009; Xie and Jiang, 2014; Ghabrial et al., 2015). With the exception of a circular single stranded (ss) DNA mycovirus from the plant pathogenic fungus *Sclerotinia sclerotiorum* (Yu et al., 2010), mycoviruses inducing hypovirulence have all single or double-stranded (ds) RNA genomes, and are included in the *Totiviridae, Chrysoviridae, Partitiviridae, Hypoviridae, Narnaviridae*, and *Reoviridae* families.

The species *Fusarium oxysporum* contains important plant-pathogens that cause a wide range of plant diseases, mostly involving a vascular wilt syndrome. Strains of *F. oxysporum* are classified in *formae speciales* on the basis of the host plant affected. *F. oxysporum* f. sp. *dianthi* is the *forma specialis* of *F. oxysporum* that infects carnation (*Dianthus caryophillus*), causing the most devastating carnation disease worldwide (Garibaldi and Gullino, 1987; Baayen, 1988). A few mycoviruses have been described infecting different *F. oxysporum* strains, but none of them could be associated with the induction of hypovirulence in its fungal host (Kilic and Griffin, 1998; Sharzei et al., 2007). Recently, we have described Fusarium oxysporum f. sp. dianthi virus 1 (FodV1), the first hypovirulence-inducing mycovirus described in the species *F. oxysporum*. FodV1 was detected infecting isolate *Fod* 116 of *F. oxysporum* f. sp. *dianthi* (Lemus-Minor et al., 2015). FodV1 consists of four dsRNA segments of 3,555 bp (dsRNA1), 2,809 bp (dsRNA2), 2,794 bp (dsRNA3), and 2,646 bp (dsRNA4), respectively. FodV1 dsRNA1 and dsRNA3 encode a RNA-dependent RNA polymerase (RdRp) and a coat protein, respectively; dsRNA2 and dsRNA4 encode hypothetical proteins with unknown functions (Lemus-Minor et al., 2015). Phylogenetic analysis with the aminoacid sequences identifies FodV1 as a member of the *Chrysoviridae* family, and places it in the same branch as the so-called “chryso-like” mycoviruses, all of them related with the alteration of phenotypic traits in their hosts (Darissa et al., 2012; Lee et al., 2014; Lemus-Minor et al., 2015). The effect of FodV1 on the phenotype of the fungus has been analyzed using two versions of isolate *Fod* 116: the original one infected with a very high titer of the virus (116V^+^), and another one with a residual titer obtained by single conidia selection (116V^−^). Results obtained by comparison of both versions evidenced a significant effect of FodV1 on all the phenotypic traits analyzed (Lemus-Minor et al., 2018). Presence of a high titer of FodV1 in isolate *Fod* 116V^+^ modified the morphology and decreased the radial growth of the colony on solid medium, reduced the conidiation in liquid medium, and lowered the virulence against carnation of its fungal host. It has also been demonstrated that FodV1 can be transferred to another isolate in the laboratory by hyphal anastomosis (Lemus-Minor et al., 2019). To prove this, we have used the originally infected strain *Fod* 116V^+^ as a donor, and strain *Fod* 77, a vegetatively compatible virus-free strain that had previously been transformed with a hygromycin resistance gene (*Fod* 77Hyg^R^), as a recipient. Results obtained showed that FodV1 not only was transferred to the recipient isolate, but also accumulated in it at a similarly high level. Comparative analysis of isolates *Fod* 77Hyg^R^ (virus-free) and *Fod* 77Hyg^R^V^+^ (virus-infected) proved that FodV1 induces similar phenotypic alterations in the new infected isolate that those described in the original infected isolate *Fod* 116V^+^ (Lemus-Minor et al., 2019).

Previous fungal isolation assays using carnation plants inoculated with V^+^ and V^−^ versions of isolate *Fod* 116 suggested that hypovirulence caused by mycovirus FodV1 could be associated to a delay in the progress of plant colonization by the virus-infected isolate (Lemus-Minor et al., 2018), but this point has not yet been experimentally supported. Using confocal laser scanner microscopy (CLSM) and GFP-labeled strains of *F. oxysporum* f. sp. *dianthi* infected and not infected with the Fusarium oxysporum f. sp. dianthi virus 1 (FodV1), respectively, we have investigated the effect of mycovirus FodV1 on the ability of the fungal isolate to colonize the plant. In addition, as the success in using mycoviruses for the control of phytopathogenic fungi depends on their efficient transmission among isolates in the natural fungal populations, we have analyzed the efficiency of FodV1-viral dispersion by examining the incidence of FodV1 viral-infections in a wide collection of *F. oxysporum* f. sp. *dianthi* isolates from the same geographic area as the original infected isolate 116V^+^.

## Materials and Methods

### Fungal Isolates and Culture Conditions

Mycovirus FodV1 was first identified in isolate *Fod* 116 (116V^+^), a race 2 strain of *F. oxysporum* f. sp. *dianthi* obtained in 2008 from a diseased carnation plant collected in the carnation growing area of Chipiona (Cádiz province, Spain) (Gómez-Lama Cabanás et al., 2012; Lemus-Minor et al., 2015). To study the prevalence of FodV1-viral infections, a collection of 221 *F. oxysporum* f. sp. *dianthi* isolates obtained between 2008 and 2012 from plants and soils in the geographic area of Cádiz and Seville provinces has been analyzed (Table 1). To perform the histologic analyses by CLSM, isogenic virus-free (V^−^) and virus-infected (V^+^) GFP-labeled strains of isolate *Fod* 77 were obtained (see below). All strains were stored as conidial suspensions at −80°C in glycerol, and propagated in potato dextrose broth (PDB) liquid medium with agitation (125 rpm) at 25°C in the dark.

**Table 1:** *Fusarium oxysporum* f. sp. *dianthi* isolates analyzed for the presence of FodV1 viral infection.

**Isolate (s)^a^**	**Geographic origin^b^**	**Source**	**Year**	**Race group assignation by PCR pattern^c^**
*Fod* 108, 110, 111, 120, 121	Chipiona(Ca)	Plant	2008	R1t
*Fod* 112, 113, 114, 117	Chipiona(Ca)	Plant	2008	R2I
*Fod* 118, 119	Chipiona(Ca)	Plant	2008	R2II
*Fod* 115	Chipiona(Ca)	Plant	2008	–
*Fod* 124, 127, 132, 134, 136, 138	Chipiona(Ca)	Plant	2009	R2I
*Fod* 122, 128, 130, 140, 142	Chipiona(Ca)	Plant	2009	R1t
*Fod* 183, 185, 187	Chipiona(Ca)	Plant	2010	R2I
*Fod* 181, 189, 191	Chipiona(Ca)	Plant	2010	R1t
*Fod* 179, 195,	Chipiona(Ca)	Soil	2010	R2II
*Fod* 197	Chipiona(Ca)	Soil	2010	R1t
*Fod* 144, 146, 148, 150, 158, 160, 162, 164	La Colonia(Se)	Plant	2010	R2I
*Fod* 152, 154	Lebrija(Se)	Plant	2010	R1t
*Fod* 156	Lebrija(Se)	Plant	2010	R2II
*Fod* 223, 225, 227, 229, 231, 233, 235, 237, 239, 241, 245, 247, 249, 251, 253	Chipiona(Ca)	Plant	2011	R2I
*Fod* 200, 201, 203, 205	Chipiona(Ca)	Plant	2011	R1t
*Fod* 207	Chipiona(Ca)	Soil	2011	R2I
*Fod* 256, 258, 259, 260, 261, 264, 265, 267, 269, 272, 275, 281	Chipiona(Ca)	Soil	2011	R2II
*Fod* 270, 280	Chipiona(Ca)	Soil	2011	R1t
*Fod* 211	Lebrija(Se)	Plant	2011	R2I
*Fod* 207, 210, 213, 215, 217, 219, 221	Lebrija(Se)	Plant	2011	R2I
*Fod* 327, 328, 329, 330, 331, 332, 333, 334, 335, 336, 341.1, 357, 358, 359, 360, 361, 364, 368, 373.2, 433, 434, 435, 453	Chipiona(Ca)	Plant	2012	R2I
*Fod* 365, 452	Chipiona(Ca)	Plant	2012	R2II
*Fod* 341.2, 342, 343, 344, 345, 346, 347, 348, 349, 350, 351, 363, 366, 367, 371, 372, 373.1, 471	Chipiona(Ca)	Plant	2012	R1t
*Fod* 337, 338, 339, 340, 369, 370, 375, 437, 438, 439, 442, 443, 444, 445, 446, 451, 469	Chipiona(Ca)	Soil	2012	R2II
*Fod* 362, 376, 440, 441, 447, 448, 450, 454, 459, 463, 476	Chipiona(Ca)	Soil	2012	R2II
*Fod* 352, 353, 354, 355, 356, 374, 449, 455, 456, 457, 458, 460, 461, 462, 464, 465, 466, 467, 468, 470, 472, 473, 474, 475, 477, 478, 479, 480, 481, 482, 483, 484, 485, 486, 487	Chipiona(Ca)	Soil	2012	R1t
*Fod* 383, 384, 385, 386, 387, 388	Lebrija(Se)	Plant	2012	R2I
*Fod* 377, 378, 379, 380, 381, 382, 391, 392, 393, 394	Lebrija(Se)	Plant	2012	R1t
*Fod* 400, 402	Lebrija(Se)	Soil	2012	R2I
*Fod* 389, 390, 395, 403, 404, 405	Lebrija(Se)	Soil	2012	R2II
*Fod* 396, 397, 398, 399, 401	Lebrija(Se)	Soil	2012	R1t

a *Isolates had been previously characterized to race-group by specific-PCR amplifications, as described in Gómez-Lama Cabanás et al. (2012)*.

*dsRNA from each isolate was purified by cellulose column chromatography and analyzed by RT-PCR using specific primers FodV1f/FodV1r directed to the RdRp sequence of mycovirus FodV1 (Lemus-Minor et al., 2018)*.

b *Ca, Cádiz province; Se, Sevilla province*.

c *Race-group assignation by molecular markers. R1t, race 1molecular group type; R2I, race 2 molecular group I; R2II, race 2 molecular group II*.

*(–) Unknown data*.

### dsRNA Extraction and FodV1 Detection

Infection by mycovirus FodV1 was determined in dsRNA extracts. To obtain the dsRNA-enriched extracts, 3 mg of mycelia were ground in liquid nitrogen, suspended in 2 x STE buffer (50 mM Tris-HCl, pH 7.0, 0.1 M NaCl, 1 mM EDTA) and 10% SDS, and the total nucleic acids were extracted with phenol. The extracts were then purified by cellulose column chromatography (Valverde et al., 1990), and visualized by electrophoresis on 1% agarose gels stained with RedSafe Nucleic Acid Staining Solution (iNtRON Biotechnology, Seongnam-si Gyeonggi-do, Korea). Presence of FodV1-dsRNA in the extracts was detected by retrotranscription (RT) followed by a polymerase chain reaction (PCR) using primers directed to the RNA-dependent RNA polymerase (RdRp) sequence of FodV1. Primers FodV1RT (5′-GGGTGGAGACTTGCGATCAT-3′) and FodV1F/FodV1R (5′-GGCCTGCTGACCCCCGACATAG-3′/5′-GACCCGAGGCAGCTGAACCCAATA-3′) were used for the RT and the PCR, respectively. RT reactions were performed from 2 μL of dsRNA extract using the enzyme NZY M-MuLV reverse transcriptase (NZYTech). PCR amplifications were performed using 2 μL of the cDNA synthesized, and the enzyme GoTaq^®^ DNA polymerase (Promega Corporation, Madison, WI USA).

RT and PCR amplification conditions were as described in Lemus-Minor et al. (2018). The products of the RT-PCR reactions were analyzed by electrophoresis on 1.5% agarose gels, purified from the gel and sequenced, and the sequences were analyzed by homology with the RdRp sequence of FodV1 using the MegAling program (DNASTAR Lasergene 15 software package).

### Obtention of a GFP-Expressing Version of Isolate *Fod* 77 and Transference of Mycovirus FodV1

To generate a version of strain *Fod* 77 constitutively expressing a green fluorescent gene (GFP), an *Agrobacterium tumefaciens*-mediated transformation (ATMT) method was used. To obtain a conidial suspension, strain *Fod* 77 was grown in YEPS (1% yeast extract, 2% bactopeptone, 2% sucrose) for 4 days at 25°C under 200 rpm shaking. Conidia were harvested by filtration through sterile Miracloth assembled in funnels and centrifuged at 4,000 × g for 10 min. The conidia concentration was determined using a hemocytometer and diluted to a final concentration of 10^7^ conidia/ml. A binary vector containing a *hygR* marker and a green fluorescent protein (GFP) cassette (Sarmiento-Villamil et al., 2018) was generated using a variant of the OSCAR method (Paz et al., 2011; Gold et al., 2017), and introduced into the AGL-1 *Agrobacterium tumefaciens* strain by electroporation. ATMT of the conidial suspension of *Fod* 77 was performed as described by Dobinson et al. (2004) with minor modifications. Transformants were selected on PDA containing hygromycin B (75 μg/ml). To confirm the integration of the selective marker in the genome, a PCR analysis of the transformants was performed with primers Hyg-F (5′-AAAGCCTGAACTCACCGCGACG-3') and Hyg-R (5′-AGCGCGTCTGCTGCTCCATAC-3′), which amplified a 736 bp region of the *hygR* gene.

To generate the mycovirus-infected GFP-tagged strain, FodV1 was transferred from isolate *Fod* 116V^+^ to isolate *Fod* 77-GFP by hyphal anastomosis. For that, mycelial plugs of the mycovirus donor and recipient strains were transferred to a PDA plate, placing them about 1–1.5 cm apart. Plates were then incubated for 10 days at 25°C to allow strains come into contact and exchange genetic material. Mycelial plugs were then taken from different points of the area of contact of both strains, and transferred to fresh PDA plates containing hygromicin B (75 μg/ml) to selectively grow the recipient strain. Selection plates were incubated for 7 days and then flasks with 100 ml of PDB were inoculated with 2–3 fragments of fungal tissue from each plate. After 4 days of growth, mycelium was collected from the liquid cultures by filtration, and ground to fine powder under liquid nitrogen. Samples were then subjected to chromatography on cellulose to detect presence of dsRNA viral molecules as described previously.

### Colonization and Virulence Bioassays

One-month cuttings of susceptible carnation cultivar Candela were inoculated by root-deeping in a suspension of conidia (10^6^ conidia/ml) of the GFP-tagged isolate *Fod* 77V^+^ or isolate *Fod* 77V^−^. Candela cuttings treated with water (not inoculated) were used as controls. Obtention of fungal inoculum, inoculation of carnation cuttings, and greenhouse conditions, were as described in Gómez-Lama Cabanás et al. (2012). Two bioassays were carried out simultaneously, using 35 plants (replicates) per treatment for each bioassay. A set of 8 plants per treatment were maintained throughout a total period of 40 days to evaluate the progress in the disease severity symptoms. Fusarium wilt symptoms were evaluated every 2 days using a scale of disease from 0 (no symptoms) to 5 (dead plant). Disease severity values were used to calculate the percentage of the standardized area under the disease progress curve (sAUDPC). Analysis of variance (ANOVA) was used to analyze sAUDPC, and significant differences among means for disease severity values with each isolate were determined using the Fisher's least significant difference (LSD P ≤ 0.05). ANOVA analyses were performed using the GraphPad Prism 6 program.

One week before ending the bioassay, a plant inoculated with each V^−^ and V^+^ *Fod* 77 strain was used to isolate the fungus, as described in Lemus-Minor et al. (2018). Mycelia from representatives of the fungal colonies obtained were analyzed by multiplex PCR (Gómez-Lama Cabanás et al., 2012) to confirm the *forma specialis* and race-group of the fungus, and by cellulose column chromatography to confirm presence or absence of FodV1-dsRNA.

### Microscopic Monitoring of Pathogen Progression in Carnation Plants

Plants inoculated with the GFP-labeled isolates *Fod* 77 V^−^ or *Fod* 77V^+^ were examined using confocal laser scanner microscopy (CLSM) every 24–48 h during 40 days after inoculation. Two plants per treatment were analyzed each time for CLSM analysis. Plants were carefully uprooted from the pots and the roots were dipped in tap water to be slightly washed. During the first week, root samples were taken daily and observed directly under the confocal microscope. From day 8, samples from the root crown and the internodes of the stem were also exhaustively analyzed by confocal microscopy. Around 1 cm long of stem and root crown tissue were sectioned using a Vibratome Series 1000 plus (TAAB Laboratories Equipment Ltd, Aldermarston, UK) under distilled water as described previously (Prieto et al., 2007). If colonization was observed in an internode, serial cuts of the next upper internode were also prepared to stage the colonization progress of the fungal isolate through the vascular bundles. Single confocal optical sections were thoroughly analyzed from all these vibratome sections using an Axioskop 2 MOT microscope (Carl Zeiss, Jena GmbH, Germany), which has a Krypton and an Argon lasers, controlled by LSM5 PASCAL software (Carl Zeiss). Both GFP-tagged *F. oxysporum* f. sp. *dianthi* strains infected and not infected with mycovirus FodV1, respectively, were detected using the 488 nm Argon laser emission light (detection at 500–520 nm). The recorded images were then transferred to Zeiss LSM Image Browser version 4.0 (Carl Zeiss) for an exhaustive study. Finally, confocal stacks were composed and deeply analyzed to assess colonization of Candela root and stem tissues by *F oxysporum* f. sp. *dianthi* strains. Brightness and contrast were slightly modified in final figures using PhotoShop 10.0 software (Adobe Systems, San Jose, CA, USA).

## Results

### Tranformation of Isolate *Fod* 77 With the *gfp* Gene and Transference of Mycovirus FodV1

To obtain a *F. oxysporum* f. sp. *dianthi* strain expressing the green fluorescent protein (GFP), a binary vector containing a *hygR* cassette next to a sGFP gene under the control of the *Aspergillus nidulans* Pgdp promoter between the right and left border of the T-DNA (Sarmiento-Villamil et al., 2018) was used. This vector was used for *Agrobacterium tumefaciens*-mediated transformation of parental virus-free isolate *Fod* 77. Three of the transformants obtained following two rounds of selection on PDA plates containing hygromycin B (75 μg/ml) were selected for detailed analysis. The presence of the transgene in the selected transformants was confirmed by performing PCR amplification of a 736 bp amplicon using the primer pair specific for the selectable gene *hyg*, while no amplification was observed in wild type *Fod* 77 isolate (data not shown). Comparison of growth rates and colony morphology on PDA plates of the three transformants and the wild type *Fod* 77 showed no significant differences.

One of the three transformants was selected as virus-free version of the GFP-tagged *Fod* 77, which was named *Fod* 77-GFP. Before transferring mycovirus FodV1 to *Fod* 77-GFP, we performed a pathogenicity test to discard that the integration of the T-DNA carrying the *gfp* gene had any effect on the virulence of the fungal isolate. To do that, we inoculated the wild type strain *Fod* 77 and the GFP expressing strain *Fod* 77-GFP on susceptible carnation cultivars Candela and Pink Bijou, and compared the progress of disease severity symptoms. Results obtained confirmed that *Fod* 77-GFP was as virulent as the parental wild type (Supplementary Table 1).

To generate isogenic lines with and without viral infection, the mycovirus FodV1 was transferred from isolate *Fod* 116V^+^ to the compatible isolate *Fod* 77-GFP by hyphal anastomosis. FodV1 donor strain *Fod* 116 and recipient strain *Fod* 77-GFP were co-cultivated on PDA plates. All the samples taken from different points of the area in contact between both strains and subcultured on PDA with hygromicin were found to contain the virus. One of these samples was chosen for further work as the FodV1-infected version of the GFP-tagged *Fod* 77.

### Mycovirus FodV1 Decreases the Speed and Modifies the Spatial Distribution of the Plant Colonization by Its Fungal Host

A detailed microscopic study of Candela root and stem tissues by CLSM was carried out to reveal the progress of vascular colonization by the GFP-labeled strains *Fod* 77-virus free (V^−^) and *Fod* 77-virus infected (V^+^). Candela carnation plants were inoculated with both *Fod* 77 strains, respectively, and samples were taken every 24–48 h and used for CLSM analyses. A set of the inoculated carnation plants were maintained throughout the whole bioassay and used to register the progress of external disease severity symptoms.

**Figure 1:**
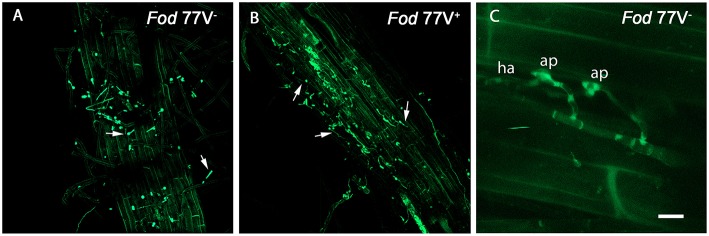
External colonization of carnation roots by the virus-free (V^−^) and the virus-infected (V^+^) GFP-strains of *Fusarium oxysporum f. sp. dianthi* isolate 77. Images were taken using a confocal laser scanning microscope and intact roots sampled at two **(A,B)** and three **(C)** days after inoculation with the virus-free strain V^−^ **(A,C)** or the virus-infected strain V^+^ **(B)**. **(A,B)** Superficial colonization of adventitious roots by the virus-free **(A)** and the virus-infected **(B)** strains showing single and germinating conidia (arrowed). **(C)** Event of penetration of the root epidermis cells showing the formation of apressoria (ap) on the cell surface, and haustoria (ha) inside the epidermal cell. Scale bar = 50 μm **(A,B)** and 5 μm **(C)**.

Weak autofluorescence from cell walls was always observed using CLSM, but did not interfere with detection of the GFP-tagged pathogen, even helping to image plant tissues and cells morphology and avoiding extra tissue staining. No autofluorescent native microorganisms were detected on/in Candela tissues at any time. Observation of intact roots between 1 and 7 days post inoculation (dpi) for both treatments showed that initial stages of carnation root colonization were similar in plants inoculated with the virus-free (V^−^) or the virus-infected (V^+^) *Fod* 77-GFP isolate. *F. oxysporum* f. sp. *dianti* conidia were readily detected on the root surface just 1 dpi, and most of these conidia had begun to germinate at 2 dpi (Figures 1A,B). After 2–4 more days, a hank of hyphae was observed wrapping the root surface. In these early days we were even able to visualize some events of fungal penetration, identifying the formation of apressoria over the surface of the root epidermal cells and haustoria intracellularly (Figure 1C). Since penetration of epidermal root cells was detected, cross-sections of the root crown zone were examined every 48 h to detect the presence of hyphal internal colonization.

**Figure 2:**
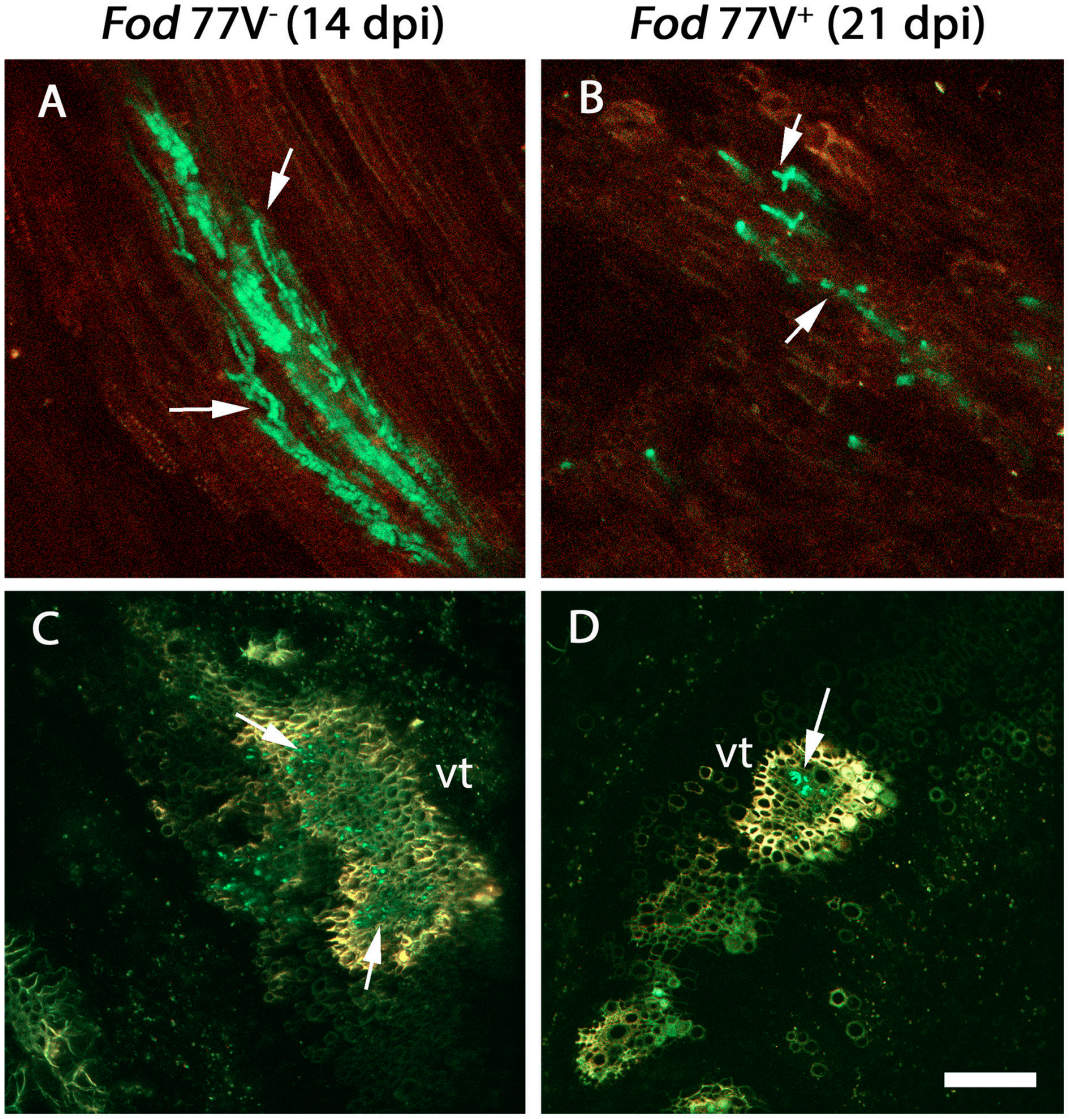
Colonization of the root crown by the virus-free (V^−^) and the virus-infected (V^+^) GFP- strains of *F. oxysporum* f. sp. *dianthi* isolate 77. **(A)** Longitudinal and **(C)** transversal root crown sections of plants inoculated with the virus-free strain V^−^ (green) 14 days after inoculation. **(B)** Longitudinal and **(D)** transversal root crown sections of plants inoculated with the virus-infected strain V^+^ (green) 21 days after inoculation. Both, the virus free and the virus-infected strain, colonized the plant vascular tissue (arrowed), but the number of vessels colonized as well as the number of hyphae inside them were lower in plants inoculated with the strain carrying the virus. vt, vascular tissue. Scale bar = 50 μm **(A,B)** and 100 μm **(C,D)**.

**Figure 3:**
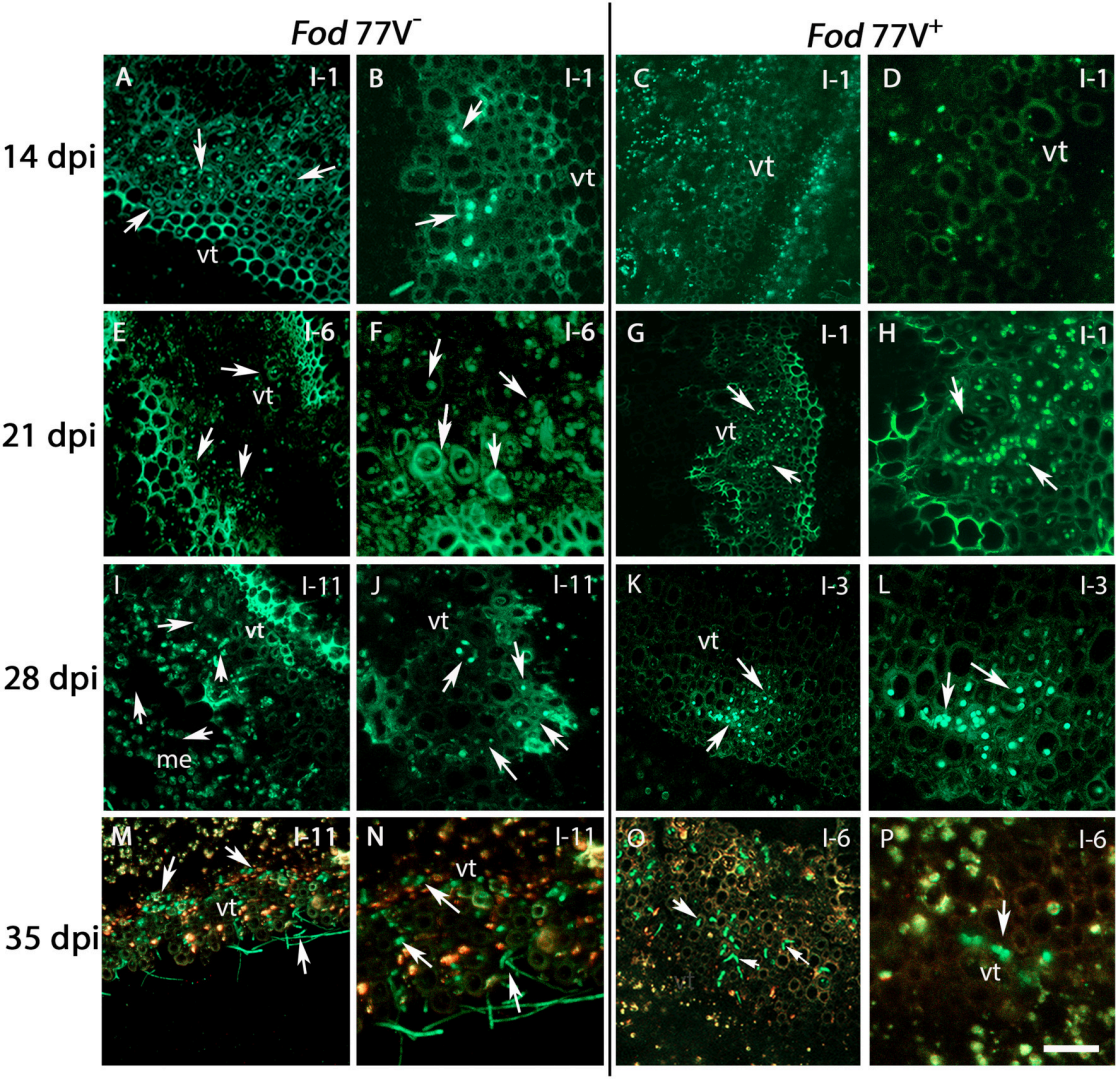
Internal colonization of stem internodes after inoculation of carnation plants with the virus-free (V^−^) and the virus-infected (V^+^) GFP-strains of *F. oxysporum* f. sp. *dianthi* isolate 77. Transversal sections of the internodes (I) were obtained at 14, 21, 28, and 35 days post inoculation (dpi), and were analyzed by confocal laser scanner microscopy. **(A,B)** Hyphae of the virus-free strain were detected in the vascular tissue of the first internode (I-1) at 14 dpi. **(C,D)** At the same sampling time (14 dpi), hyphae of the virus–infected strain were not detected yet in the same internode. **(E,F)** Stem sections of the sixth internode (I-6) showing the vascular tissue extensively colonized by hyphae of strain V^−^ at 21 dpi. **(G,H)** At the same time point, hyphae of strain V^+^ only reached the first internode (I-1). **(I,J)** Stem sections of plants inoculated with the strain V^−^ at 28 dpi showing the hyphae reaching the last stem internode (I-11) of the plant. **(K,L)** At the same sampling time (28 dpi), the strain V^+^ only reached the vascular tissue of internode 3 (I-3). **(M,N)** Extensive colonization of the vascular tissue up to the last plant internode (I-11) by the virus free strain at 35 dpi. **(O,P)** In contrast, colonization by the V^+^ strain did only reach internode 6 (I-6) at the same sampling time (35 dpi). Arrows indicate the presence of hyphae (green). vt, vascular tissue. Scale bar = 50 μm **(A**,**C**,**E**,**G**,**I**,**K**,**M,O)**, 20 μm **(B**,**D**,**F**,**H**,**J**,**L**,**N,P)**.

Root crown sections obtained at 6, 8, 10, and 12 dpi from plants inoculated with either strain *Fod* 77V^+^ or *Fod* 77V^−^ were all free of fungal colonization. Colonization in this zone was first detected at 14 dpi only in crown sections from carnation plants inoculated with the virus-free strain (Figures 2A,C). At this same sampling time some hyphae of the V^−^ strain were already slightly reaching the vascular tissue in the first internode of the stem (Figures 3A,B). In contrast, no fungal signals were detected in plants inoculated with the strain carrying the virus (V^+^) at this stage (Figures 3C,D). In fact, colonization of the root crown of plants inoculated with the strain carrying the virus was firstly detected at 21 dpi (this means 7 days later than with the strain without the virus) (Figures 2B,D). In addition, our observations also suggested that, although hyphae of both fungal strains (V^−^ and V^+^) colonized the vascular tissue in the root crown zone, the number of vessels colonized and the hyphae density inside them seemed to be always markedly lower in the plants inoculated with the strain carrying the virus (V^+^) (Figure 2).

Analysis of stem cross sections at 21 dpi evidenced a rapid progress of the colonization by the virus-free strain, already detecting the hyphae of the fungus in the internode 6 of the carnation plants (Figures 3E,F). On the contrary, at this sampling time hyphae of the strain carrying the virus were only observed in one out of four plants sampled and reaching only the first internode (Figures 3G,H). It is worthy to mention that, at this sampling time, CLSM observations were consistent with visible symptoms of the carnation plants, which were rated quite differently among both treatments. Thus, plants inoculated with the virus-free strain V^−^ showed visible symptoms of disease rated as 3 in a 0–5 scale, whereas plants inoculated with the virus-infected strain V^+^ were rated 0–1.

**Figure 4:**
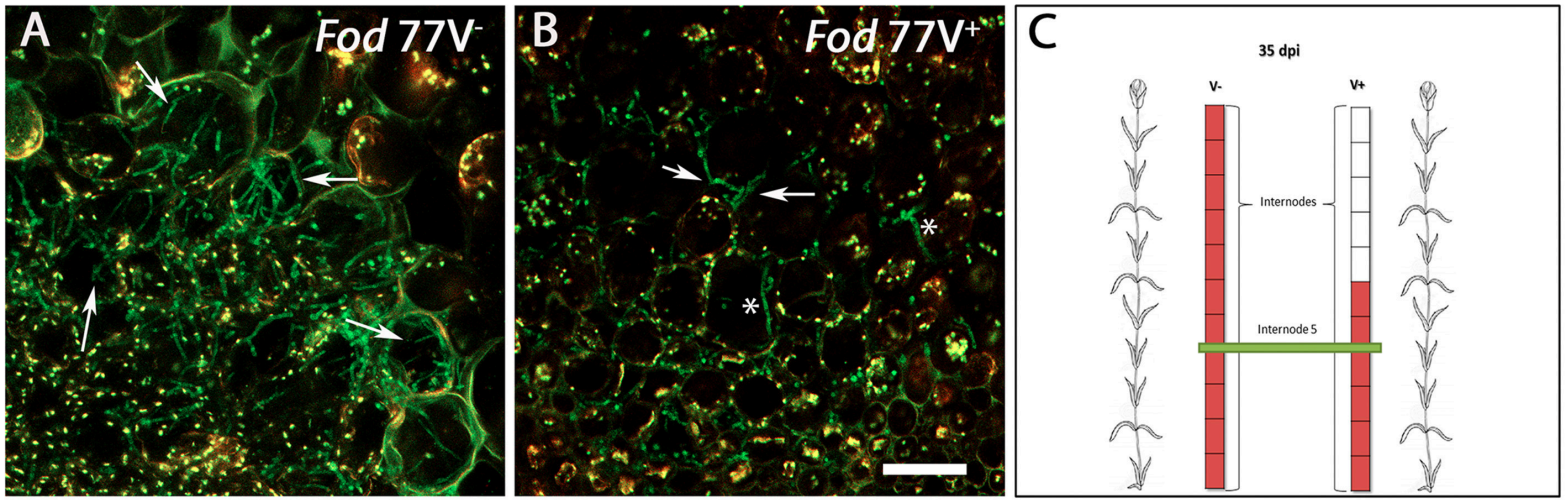
Differences in the colonization pattern of the central medulla in stem sections of plants inoculated with the virus-free (V^−^) or the virus-infected (V^+^) GFP-strain of *F. oxysporum* f. sp. *dianthi* isolate 77. Images correspond to transversal sections from the same internode (fifth internode) of plants inoculated with the virus-free V^−^ **(A)** and the virus-infected V^+^ **(B)** strains (green), 35 days after inoculation. **(A)** Hyphae of the virus free strain profusely colonizing the interior of the medulla cells. **(B)** Hyphae of the virus-infected strain preferentially located in the intercellular spaces of the medulla cells (arrowed) although some hyphae seem to invade the interior of the cells (asterisks). **(C)** Squematic representation of carnation plants indicating the level of colonization (red) by the virus-free and the virus-infected strains as well as the area shown in panels **A** and **B** corresponding to internode 5 (green). Scale bar represents 50 μm in **(A)** and **(B)**.

Successive observation of stem cross-sections of the sampled plants displayed a clear difference in the colonization pattern between *Fod* 77V^−^ and *Fod* 77V^+^ strains. Differences were found related to the rate of progression of the plant colonization by the fungal strains. Cross sections examined at 28 and 35 dpi showed an extensive colonization of the stem including the last internode (internode 11) in plants inoculated with the virus-free strain V^−^ (Figures 3I,J,M,N). On the contrary, plants inoculated with the virus-infected strain V^+^ only showed colonization of the vascular tissue up to internode 3 at 28 dpi (Figures 3K,L), and up to internode 6 at day 35 (Figures 3O,P). In addition to this delay in the colonization progress by the strain carrying the virus, the density of colonization (number of vessels colonized and hyphae inside each vessel) observed at these sampling times seemed to be lower in stem sections of plants inoculated with the virus-infected strain V^+^ (Figures 3I–P). As colonization progressed, both strains did also invade other plant tissues (medulla) and differences in density between both strains were detected too (Figure 4). Moreover, while hyphae of the virus-free strain V^−^ were easily detected occupying profusely the interior of the medulla cells, hyphae of the virus-infected strain V^+^ were preferentially observed in the intercellular spaces of the medulla and rarely were able to invade these cells (Figure 4).

**Figure 5:**
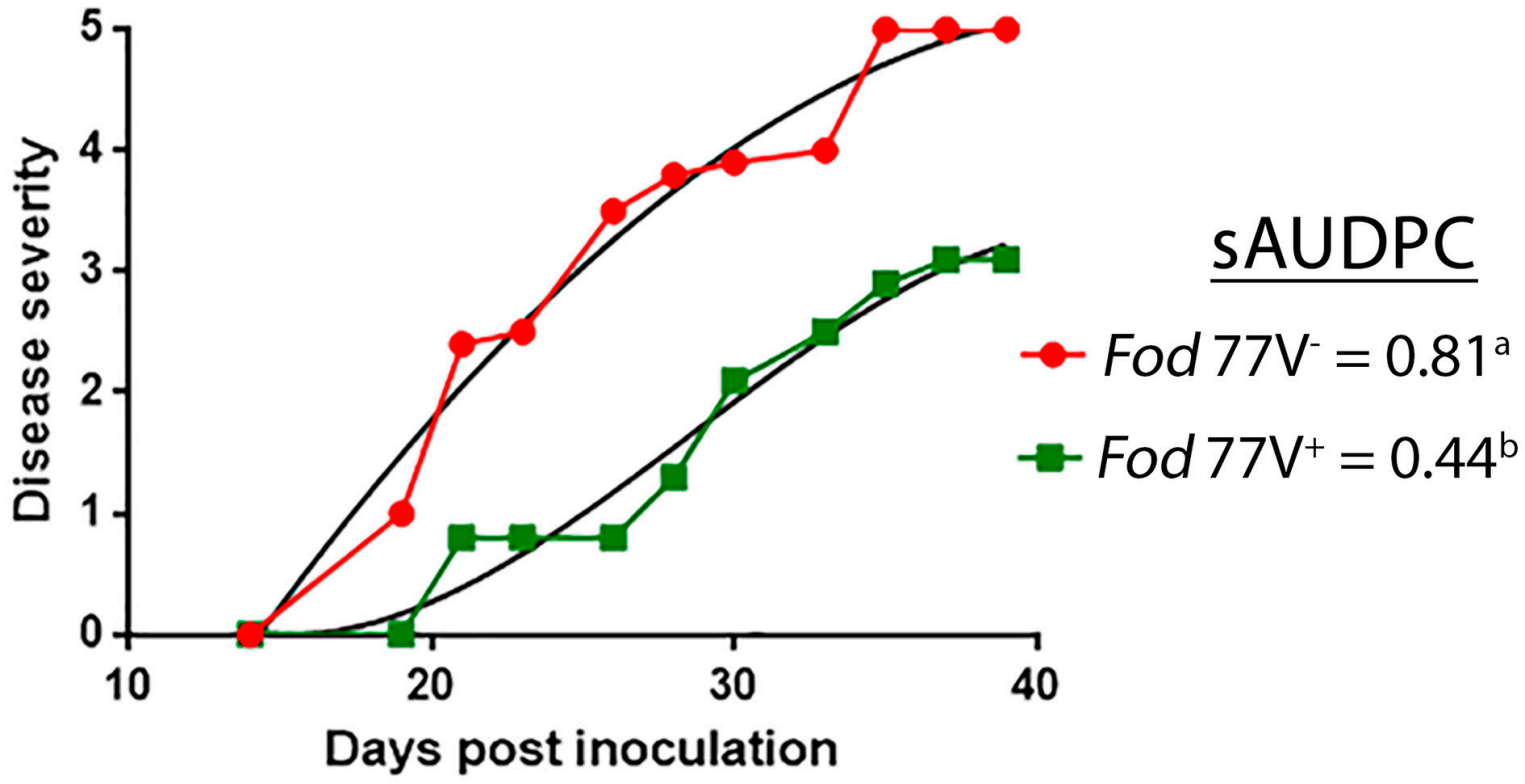
Representative graph of the progress of the disease severity symptoms. Fusarium wilt symptoms were scored for a period of 40 days, using a scale from 0 (no symptoms) to 5 (dead plant), in carnation plants inoculated with the virus-free (V^−^) or the virus-infected (V^+^) strain of *F. oxysporum* f. sp. *dianthi* isolate 77. Disease severity values were used to calculate the percentage of the standardized area under the disease progress curve (sAUDPC). Values followed by different letters are significantly different according to Fisher's least significant difference (LSD) test (*P* ≤ 0.05).

At the end of the bioassay (40 dpi), all plants inoculated with the virus-free strain were dead (disease severity = 5) whereas plants inoculated with the virus- infected strain were still alive and maintained disease severity values ≤ 3. Analysis of the disease severity data obtained during the bioassay evidenced a significant reduction of the sAUDPC when cultivar Candela was inoculated with isolate *Fod* 77V^+^ compared to isolate *Fod* 77V^−^ (Figure 5).

**Figure 6:**
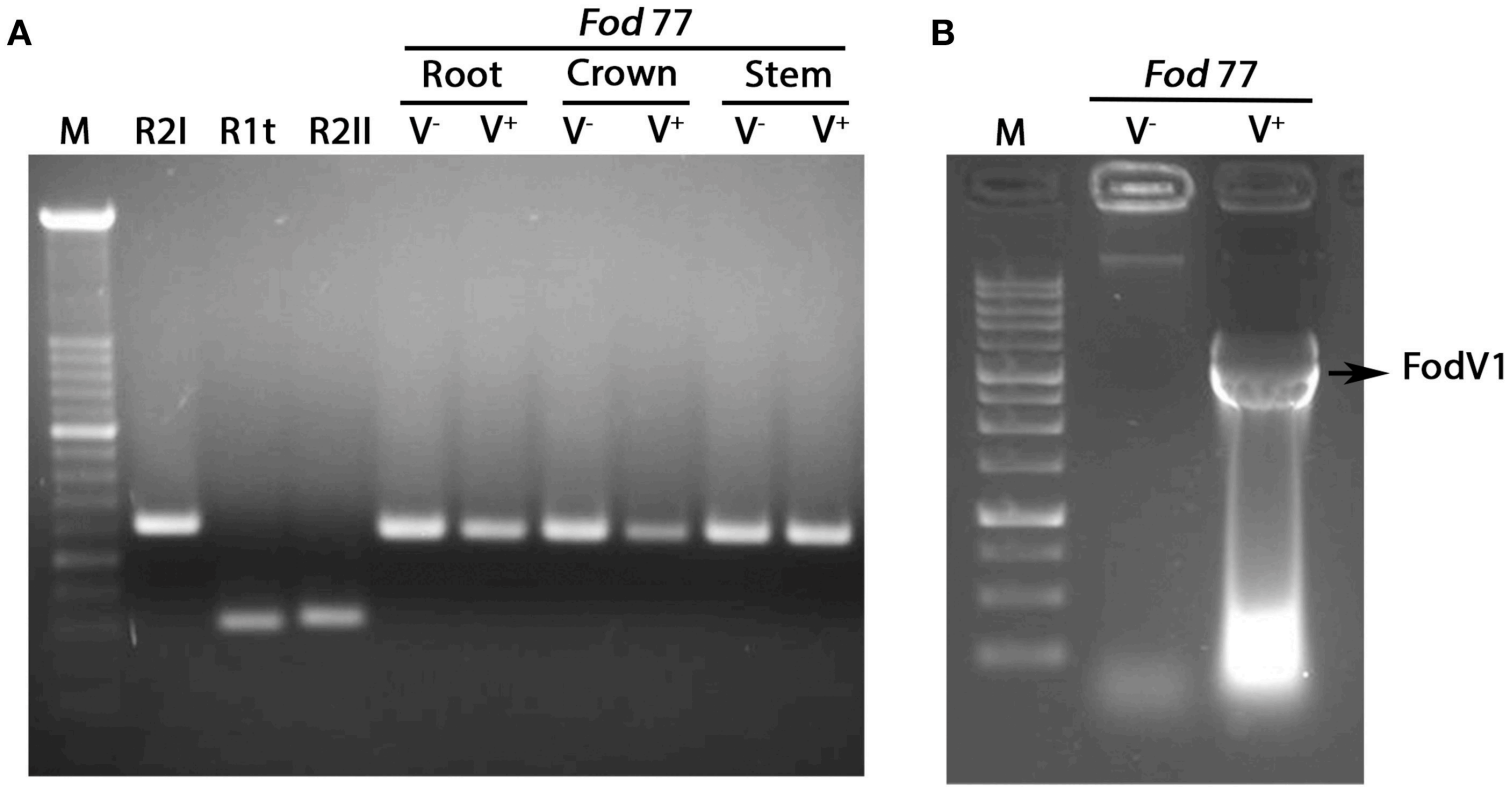
Analysis of the fungal mycelia reisolated from plants inoculated with the virus-free (V^−^) and the virus-infected (V^+^) strain of *Fusarium oxysporum* f. sp. *dianthi* isolate 77. **(A)** Amplification products obtained in multiplex-PCR reactions using specific primers for the different races-groups described in *F. oxysporum* f. sp. *dianthi* (*Fod*) and DNA from the fungal colonies recovered from root, crown, or stem of carnation plants inoculated with the virus-free strain V^−^ or the virus-infected strain V^+^ of isolate *Fod* 77. Specific primers used and conditions of the multiplex-PCR were as described in Gómez-Lama Cabanás et al. (2012). M, molecular weight marker XIV (Roche Diagnostics). R2I, R1t, and R2II, representative of the respective race-group described in *F. oxysporum* f. sp. *dianthi*. **(B)** Extracts of dsRNA obtained by cellulose column chromatography using mycelia recovered from a carnation plant inoculated with the virus-free (V^−^) or the virus-infected (V^+^) strain of isolate *Fod* 77. M, 1Kb DNA Ladder (Nippon Genetics).

Analysis of the mycelia recovered from the inoculated plants confirmed the identity (*F. oxysporum* f. sp. *dianthi* race 2 group I) and presence or absence of viral infection in each inoculated *Fod* 77 strain (Figure 6).

### Mycovirus FodV1 Has a Low Incidence in the Collection of *F. oxysporum* f. sp. *dianthi* Isolates Analyzed

Micovirus FodV1 was detected infecting isolate *Fod* 116, a race 2 isolate obtained in 2008 from a carnation plant collected in Chipiona (Cádiz, Spain). To determine the incidence of FodV1-dsRNA viral infections, a collection of 221 isolates of *F. oxysporum* f. sp. *dianthi* obtained between years 2008 and 2012 from plants and soils in the same (Cádiz) or adjacent (Sevilla) geographic area was analyzed (Table 1). All the isolates had been previously characterized to race by molecular markers (Table 1), and some of them also to vegetative compatibility group (VCG) (Gómez-Lama Cabanás and Pérez-Artés, 2014).

**Figure 7:**
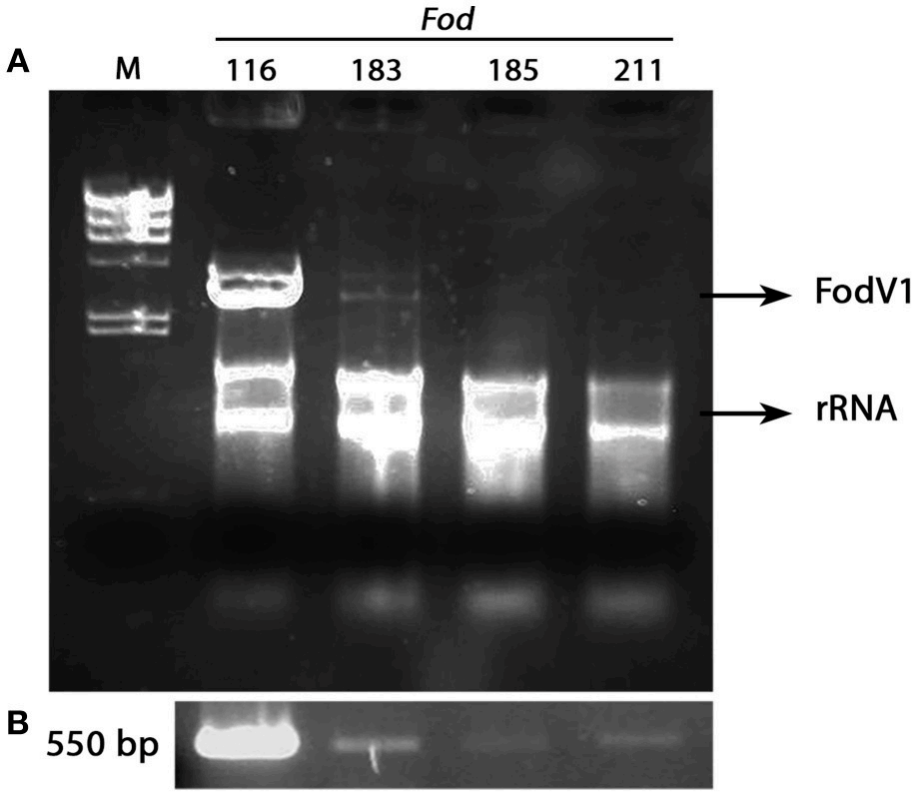
Isolates of *Fusarium oxysporum* f. sp. *dianthi* infected with mycovirus FodV1. **(A)** dsRNA-enriched extracts obtained by cellulose column chromatography from isolates *Fod* 116 (the originally infected isolate), *Fod* 183, *Fod* 185, and *Fod* 211. **(B)** Specific amplicons obtained after RT-PCR of the above dsRNA extracts using specific primers for the RdRp sequence of FodV1. M, molecular weight marker II (Roche Diagnostics) (see also Supplementary Image to Figure 7).

The dsRNA-enriched extracts were obtained by cellulose column chromatography and subjected to RT-PCR amplification using specific primers for the RdRp sequence of FodV1 (Figure 7). Direct observation after agarosa gel electrophoresis of the dsRNA extracts indicated presence of FodV1 only in isolate *Fod* 183, an isolate obtained from a carnation plant in 2010 in Chipiona (Cádiz) (Figure 7A). Subsequent RT-PCR amplification of the dsRNA extracts showed two additional isolates infected with FodV1: isolate *Fod* 185, obtained from a carnation plant in 2010 in Chipiona, and isolate *Fod* 211, obtained from a carnation plant in 2011 in Lebrija (Sevilla) (Figure 7B). All the three new virus-infected isolates were race 2 and belonged to VCG 0021.

To confirm the identity of the mycovirus detected in isolates *Fod* 183, *Fod* 185 and *Fod* 211, the amplicon obtained by RT-PCR with each isolate was purified from the agarose gel and sequenced. Comparison of these sequences with that of the corresponding fragment of the RdRp sequence of FodV1 showed a 100% homology (Supplementary Figure 1).

## Discussion

The majority of the studies on the induction of hypovirulence by mycoviruses of phytopathogenic fungi have been focused on the alteration of particular phenotypic traits (reviewed in Ghabrial et al., 2015) or on the modification of the gene expression pattern of the fungal host (Allen and Nuss, 2004; Cho et al., 2012; Lee et al., 2014), but the possible effect of the hypovirulence-inducing mycovirus on the fungal colonization pattern inside the plant has not yet been investigated. In this work, we have analyzed the effect of mycovirus FodV1 on the spatial and temporal dynamics of plant colonization by using confocal laser scanner microscopy. To perform this study, we have used two isogenic GFP-labeled strains of *F. oxysporum* f. sp. *dianthi* isolate 77: the original virus-free strain (V^−^), and a FodV1-infected strain obtained in this work (V^+^). Both strains were used to inoculate carnation cuttings of susceptible cultivar Candela.

Vascular pathogens are characterized by their ability to colonize the vascular system of the host. Colonization of the xylem vessels leads to a severe decrease in the transport of water and nutrients to the aerial parts of the plant causing its wilting and finally its death. A number of studies have shown that the first stage of plant colonization is common to different *F. oxysporum* strains and hosts, and initiate with the development of a hyphae network over the roots, followed by penetration of the root epidermis (Li et al., 2011; Jiménez-Fernández et al., 2013; Niño-Sánchez et al., 2015; Upasani et al., 2016). In this work, observation of intact roots throughout the first week after inoculation showed no appreciable differences in the root colonization pattern between the virus-free and the virus-infected strains. Conidia of both strains were observed adhered and germinating over the root surface from 1 dpi, and after 2–4 days the roots were covered by a network of hyphae, some of which appeared penetrating the root epidermis. Several works using light or confocal microscopy and other *formae speciales* of *F. oxysporum* revealed the difficulty of finding the specific structures of the fungus at the onset of root cell penetration and the impossibility to photograph them (Lagopodi et al., 2002; Jiménez-Fernández et al., 2013; Niño-Sánchez et al., 2015; Upasani et al., 2016; Pouralibaba et al., 2017). In contrast, in this work we have been able to observe the formation of specific fungal structures such as appressoria over the root surface and haustoria inside the epidermal root cell during the plant cell penetration event using CLSM and the GFP-labeled *F. oxysporum* f. sp. *dianthi* strains.

The architecture of the stem vascular system develops in the crown area, where the primordial ring of xylem vessels is formed. Independently of the strain inoculated (V^−^ or V^+^), root crown sections obtained at 6, 8, 10, and 12 dpi were all free of fungal colonization. This seems to indicate that this period of approximately 1 week from the moment of penetration of the epidermal root cells was necessary for the fungus to reach the central roots and move upward to the crown area.

From 12 dpi, subsequent observations of root crown sections marked the first difference among the virus-free and the virus-infected strains. Colonization of the root crown was first detected in plants inoculated with the virus-free strain at 14 dpi, whereas root crown of plants inoculated with the strain carrying the virus remained free of fungal colonization till 21 dpi. Comparison of crown sections at different times until 28 dpi evidenced that, in addition to the delay in the colonization time, crown of plants inoculated with the virus-infected strain always showed a notably lower density of colonization. This means that, although the hyphae of both V^−^ and V^+^ strains were observed inside the vascular tissue at the root crown zone, the number of vessels colonized and the number of hyphae inside them was lower in the case of V^+^-infected plants.

The delay observed in the time of colonization of the root crown area in plants inoculated with strain V^+^ compared to plants inoculated with strain V^−^ was maintained during the colonization of the stem. Hyphae of the virus-free strain were detected colonizing the vessels in the first internode at 14 dpi, while hyphae of the virus-infected strain reached this same zone 6 days later (21 dpi). Once the hyphae of each strain were detected infecting the first internode of the plant, the progress of colonization upwards the stem was faster in plants inoculated with the virus-free strain. Cross stem sections of plants inoculated with strain V^−^ showed fungal hyphae reaching the last internode (internode 11) at 28 dpi, whereas at the same sampling time hyphae of strain V^+^ had colonized only the third lower part of the stem (up to internode 3). Along with this difference in the speed of colonization of the vascular system upwards the stem, some other differences were observed related to the area colonized and the density of colonization reached. The number of vessels colonized and of hyphae inside each of them observed at the different sampling times was always notably lower in stem sections of plants inoculated with the virus-infected strain.

At advanced stages of the colonization, differences in the density and location of the hyphae of strains V^−^ and V^+^ were also observed in tissues other than the vascular tissue. As colonization progressed, hyphae of both strains invaded the central area of the stem (medulla), but while hyphae of the virus-free strain V^−^ were easily detected profusely occupying the interior of the medulla cells, hyphae of the virus-infected strain V^+^ were less in number and rarely were able to invade the medulla cells, being preferentially located in the intercellular spaces. This difference in the time and extent of stem colonization correlated with significant differences in the external disease symptoms so that most of the plants inoculated with strain V^−^ were dead (disease severity = 5) at 35 dpi, whereas plants inoculated with strain V^+^ maintained disease severity values ≤3 at the end of the bioassay (40 dpi).

The importance of the speed in the colonization of the vascular tissue as determinant of disease severity has been described previously in a study using two strains of *F. oxysporum* f. sp. *phaseoli* differing in virulence (Niño-Sánchez et al., 2015). These authors reported differences on the spatial and temporal dynamic of colonization of a susceptible common bean cultivar infected by a highly virulent (HV) or a weakly virulent (WV) strain. Similar to our results, the main differences between both strains were found in the temporal and spatial dynamic of crown root and hypocotyl colonization, the WV strain being a much slower and less efficient colonizer of the xylem vessels (Niño-Sánchez et al., 2015). Differences in virulence between the HV and WV strains of *F. oxysporum* f. sp. *phaseoli* have been associated to the presence and expression of some transcription factors (Alves-Santos et al., 2002a,b; Ramos et al., 2007). In this work, difference in virulence between the V^−^ and V^+^ strains of *F. oxysporum* f. sp. *dianthi* is associated to the presence of mycovirus FodV1. Therefore, it seems that in these two specific cases, regardless of the endogenous or exogenous cause, the hypovirulence is associated to a delay and restriction in the colonization of the vascular system of the plant.

Previous microscopic analysis related to plant colonization by *F. oxysporum* have been done but focused on particular host-fungus interactions (Czymmek et al., 2007; Nahalkova et al., 2008; Li et al., 2011), compatible (susceptible) or incompatible (resistant) interactions (Zvirin et al., 2010; Jiménez-Fernández et al., 2013; Lü et al., 2013; Pouralibaba et al., 2017), or related to differences in the virulence of the pathogenic strains (Niño-Sánchez et al., 2015). To the best of our knowledge, this work provides the first microscopic evidences on the effect of a hypovirulence-inducing mycovirus on the plant colonization process by its fungal host.

Another aspect analyzed in this study has been that on the incidence of FodV1-viral infections in a collection of *F. oxysporum* f. sp. *dianthi* isolates sampled between 2008 and 2012 in the geographic area where the originally infected isolate *Fod* 116 was obtained. In a previous work, we had demonstrated that FodV1 could be transferred by hyphal anastomosis to another vegetatively compatible isolate in the laboratory (Lemus-Minor et al., 2019). In *F. oxysporum* f. sp. *dianthi*, isolates of race 1 type and of race 2 belong to different VCGs, i.e., VCG 0022 for race 1 type isolates and VCG 0021 for race 2 isolates (Gómez-Lama Cabanás and Pérez-Artés, 2014). The majority of the isolates in the collection analyzed was race 2 and thus belonged to the same 0021 vegetative compatibility group than the virus-infected isolate *Fod* 116. Therefore, in theory, FodV1 should have been able to disperse among compatible hyphae in the fungal population. In spite of that, results obtained in this work demonstrate a very low incidence of FodV1 infections. Mycovirus FodV1 has been detected infecting only another three *F. oxysporum* f. sp. *dianthi* isolates. In addition to the *vic* genes that regulate hyphal anastomosis, the host genetic background has been shown to affect horizontal virus transmission (Cortesi et al., 2001; Choi et al., 2012; Zamora et al., 2015). Although all race 2 isolates are in VCG 0021, we have evidence that some isolates in a same VCG show a reduced compatibility, that is, they can anastomose with a few but not all the isolates in the VCG (Gómez-Lama Cabanás and Pérez-Artés, 2014). The naturally infected isolate *Fod* 116 could be one of those isolates with reduced compatibility, and this fact could have limited the dispersion of the mycovirus. Another factor that could be contributing to the very low dispersion rate of FodV1 is the efficiency in vertical transmission through asexual spores. Previous studies carried out with the original virus-infected isolate *Fod* 116V^+^ and the “in laboratory” virus-infected isolate *Fod* 77V^+^ showed that efficiency in vertical transmission depends on the isolate considered (Lemus-Minor et al., 2019). Thus, while isolate *Fod* 116V^+^ showed a relatively low efficiency (24%), the efficiency of isolate *Fod* 77V^+^ was 100%. This low efficiency of vertical virus-transmission showed by isolate *Fod* 116V^+^ could be contributing to restrict the presence of new virus-infected hyphae of this isolate in the population. In this way, a low density of virus-infected hyphae would decrease the probability of contact between infected and non-infected hyphae and would be limiting the dispersion of the viral infection. This evidence supports the hypothesis that selection of a specific virus-infected isolate could influence transmission and dissemination of hypovirulence (Lee et al., 2014; Lemus-Minor et al., 2019).

Finally, we must take an additional factor into consideration. From the collection analyzed, most (127) of the isolates were obtained from plants with disease symptoms, while 94 isolates were obtained from soil samples. Considering the reduction in the disease severity symptoms associated to the viral infection, this bias in the sampling could also be skewing the data of the prevalence of viral infection in the population. To avoid this inconvenient, it should be necessary to make a more exhaustive analysis with isolates collected exclusively from soil samples.

To the best of our knowledge, this work provides the first microscopic evidence about the effect of a hypovirulence-inducing mycovirus on the plant colonization process, and supplies data that reinforce the idea that the choice of the appropriate isolate is key to achieve an efficient dissemination of a viral infection in a fungal population.

## Data Availability

All datasets generated for this study are included in the manuscript and/or the supplementary files.

## Author Contributions

EP-A, AT-T, and PP conceived and designed the experiments. MC and MG-P obtained the GFP-tagged V^+^ and V^−^ *Fod* strains. PP and AT-T performed the microscopic analysis. EP-A, AT-T, and PP analyzed the data. EP-A and PP wrote the paper.

### Conflict of Interest Statement

The authors declare that the research was conducted in the absence of any commercial or financial relationships that could be construed as a potential conflict of interest.

## References

[B1] Allen T. D. Nuss D. L. (2004). Specific and common alterations in host gene transcript accumulation following infection of the chestnut blight fungus by mild and severe hypoviruses. J. Virol. 78, 4145–4155. 10.1128/JVI.78.8.4145-4155.2004 15047830 PMC374289

[B2] Alves-Santos F. M. Cordeiro-Rodrigues L. Sayagués J. M. Martín-Domínguez R. García-Benavides P. Crespo M. C. (2002b). Pathogenicity and race characterization of *Fusarium oxysporum* f. sp. phaseoli isolates from Spain and Greece. Plant Pathol. 51, 605–611. 10.1046/j.1365-3059.2002.00745.x

[B3] Alves-Santos F. M. Ramos B. García-Sánchez M. A. Eslava A. P. Díaz-Mínguez J. M. (2002a). A DNA-based procedure for in planta detection of *Fusarium oxysporum* f. sp. phaseoli. Phytopathology 92, 237–244. 10.1094/PHYTO.2002.92.3.237 18943994

[B4] Baayen R. P. (1988). Fusarium Wilt of Carnation. Disease Development, Resistance Mechanism of the Host and Taxonomy of the Pathogen. thesis, University of Utretcht, Holland.

[B5] Cho W. K. Yu J. Lee K. M. Son M. Min K. Lee Y. W.. (2012). Genome-wide expression profiling shows transcriptional reprogramming in *Fusarium graminearum* by *Fusarium graminearum* virus 1-DK21 infection. BMC Genomics 13:173. 10.1186/1471-2164-13-173 22559730 PMC3478160

[B6] Choi G. H. Dawe A. L. Churbanov A. Smith M. L. Milgroom M. G. Nuss D. L. (2012). Molecular characterization of vegetative incompatibility genes that restrict hypovirus transmission in the chestnut blight fungus *Cryphonectria parasitica*. Genetics 190, 113–127. 10.1534/genetics.111.133983 22021387 PMC3249360

[B7] Cortesi P. McCulloch C. E. Song H. Lin H. Milgroom M. G. (2001). Genetic control of horizontal virus transmission in the chestnut blight fungus, *Cryphonectria parasitica*. Genetics 159, 107–118. 11560890 10.1093/genetics/159.1.107 PMC1461798

[B8] Czymmek K. J. Fogg M. Powell D. H. Sweigard J. Park S. Y. Kang S. (2007). *In vivo* time-lapse documentation using confocal and multi-photon microscopy reveals the mechanisms of invasion into the Arabidopsis root vascular system by *Fusarium oxysporum*. Fungal Genet. Biol. 44, 1011–1023. 10.1016/j.fgb.2007.01.012 17379550

[B9] Darissa O. Adam G. Schafer W. (2012). A dsRNA mycovirus causes hypovirulence of *Fusarium graminearum* to wheat and maize. Eur. J. Plant Pathol. 134, 181–189. 10.1007/s10658-012-9977-5

[B10] Dobinson K. F. Grant S. J. Kang S. (2004). Cloning and targeted disruption, via *Agrobacterium tumefaciens*-mediated transformation, of a trypsin protease gene from the vascular wilt fungus *Verticillium dahliae*. Curr. Genet. 45, 104–110. 10.1007/s00294-003-0464-6 14618375

[B11] Garibaldi A. Gullino M. L. (1987). Attempts of biocontrol of Fusarium-wilt of carnation in Italy. Phytopathology 77, 1721–1721.

[B12] Ghabrial S. A. Caston J. R. Jiang D. Nibert M. L. Suzuki N. (2015). 50-plus years of fungal viruses. Virology 479–480, 356–368. 10.1016/j.virol.2015.02.034 25771805

[B13] Ghabrial S. A. Suzuki N. (2009). Viruses of plant pathogenic fungi. Annu. Rev. Phytopathol. 47, 353–384. 10.1146/annurev-phyto-080508-081932 19400634

[B14] Gold S. E. Paz Z. García-Pedrajas M. D. Glenn A. E. (2017). Rapid deletion production in fungi via agrobacterium mediated transformation of OSCAR deletion constructs. J. Vis. Exp. e55239. 10.3791/55239 28654073 PMC5608391

[B15] Gómez-Lama Cabanás C. Pérez-Artés E. (2014). New evidence of intra-race diversity in *Fusarium oxysporum* f. sp. *dianthi* populations based on Vegetative Compatibility Groups. Eur. J. Plant Pathol. 139, 445–451. 10.1007/s10658-014-0412-y

[B16] Gómez-Lama Cabanás C. Valverde-Corredor A. Pérez-Artés E. (2012). Molecular analysis of Spanish populations of *Fusarium oxysporum f. sp dianthi* demonstrates a high genetic diversity and identifies virulence groups in races 1 and 2 of the pathogen. Eur. J. Plant Pathol. 132, 561–576. 10.1007/s10658-011-9901-4

[B17] Jiménez-Fernández D. Landa B. B. Kang S. Jiménez-Díaz R. M. Navas-Cortes J. A. (2013). Quantitative and microscopic assessment of compatible and incompatible interactions between chickpea cultivars and *Fusarium oxysporum f*. sp. ciceris races. PLoS ONE 8:e61360. 10.1371/journal.pone.0061360 23613839 PMC3629054

[B18] Kilic O. Griffin G. J. (1998). Effect of dsRNA-containing and dsRNA-free hypovirulent isolates of *Fusarium oxysporum* on severity of *Fusarium* seedling disease of soybean in naturally infested soil. Plant Soil 201, 125–135. 10.1023/A:1004319614390

[B19] Lagopodi A. L. Ram A. F. Lamers G. E. Punt P. J. Van den Hondel C. A. Lugtenberg B. J.. (2002). Novel aspects of tomato root colonization and infection by *Fusarium oxysporum f*. sp. radicis-lycopersici revealed by confocal laser scanning microscopic analysis using the green fluorescent protein as a marker. Mol. Plant Microbe Interact. 15, 172–179. 10.1094/MPMI.2002.15.2.172 11878320

[B20] Lee K. M. Cho W. K. Yu J. Son M. Choi H. Min K.. (2014). A comparison of transcriptional patterns and mycological phenotypes following infection of *Fusarium graminearum* by four mycoviruses. PLoS ONE 9:e100989. 10.1371/journal.pone.0100989 24964178 PMC4071046

[B21] Lemus-Minor C. G. Canizares M. C. García-Pedrajas M. D. Pérez-Artés E. (2015). Complete genome sequence of a novel dsRNA mycovirus isolated from the phytopathogenic fungus *Fusarium oxysporum f. sp. dianthi*. Arch. Virol. 160, 2375–2379. 10.1007/s00705-015-2509-9. 26138558

[B22] Lemus-Minor C. G. Canizares M. C. García-Pedrajas M. D. Pérez-Artés E. (2018). *Fusarium oxysporum f. sp. dianthi* virus 1 accumulation is correlated with changes in virulence and other phenotypic traits of its fungal host. Phytopathology 108, 957–963. 10.1094/PHYTO-06-17-0200-R 29516772

[B23] Lemus-Minor C. G. Cañizares M. C. García-Pedrajas M. D. Pérez-Artés E. (2019). Horizontal and vertical transmission of the hypovirulence-associated mycovirus *Fusarium oxysporum f. sp. dianthi* virus 1. Eur. J. Plant Pathol 153, 645–650. 10.1007/s10658-018-1554-0

[B24] Li C. Chen S. Zuo C. Sun Q. Ye Q. Yi G. (2011). The use of GFP-transformed isolates to study infection of banana with *Fusarium oxysporum f. sp. cubense* race 4. Eur. J. Plant Pathol. 131, 327–340. 10.1007/s10658-011-9811-5

[B25] Liu S. Xie J. Cheng J. Li B. Chen T. Fu Y.. (2016). Fungal DNA virus infects a mycophagous insect and utilizes it as a transmission vector. Proc. Natl. Acad. Sci. U.S.A. 113, 12803–12808. 10.1073/pnas.1608013113 27791095 PMC5111676

[B26] Lü G. Guo S. Zhang H. Geng L. Martyn R. D. Xu Y. (2013). Colonization of *Fusarium* wilt-resistant and susceptible watermelon roots by a green-fluorescent-protein-tagged isolate of *Fusarium oxysporum* f.sp. niveum. J. Phytopathol. 162, 228–237. 10.1111/jph.12174

[B27] Marzano S. L. Nelson B. D. Ajayi-Oyetunde O. Bradley C. A. Hughes T. J. Hartman G. L.. (2016). Identification of diverse mycoviruses through metatranscriptomics characterization of the viromes of five major fungal plant pathogens. J. Virol. 90, 6846–6863. 10.1128/JVI.00357-16. 27194764 PMC4944287

[B28] Nahalkova J. Fatehi J. Olivain C. Alabouvette C. (2008). Tomato root colonization by fluorescent-tagged pathogenic and protective strains of *Fusarium oxysporum* in hydroponic culture differs from root colonization in soil. FEMS Microbiol. Lett. 286, 152–157. 10.1111/j.1574-6968.2008.01241.x 18657114

[B29] Niño-Sánchez J. Tello V. Casado-Del Castillo V. Thon M. R. Benito E. P. Díaz-Mínguez J. M. (2015). Gene expression patterns and dynamics of the colonization of common bean (*Phaseolus vulgaris L*.) by highly virulent and weakly virulent strains of *Fusarium oxysporum*. Front. Microbiol. 6:234. 10.3389/fmicb.2015.00234 25883592 PMC4383042

[B30] Paz Z. García-Pedrajas M. D. Andrews D. L. Klosterman S. J. Baeza-Montanez L. Gold S. E. (2011). One step construction of Agrobacterium-Recombination-ready-plasmids (OSCAR), an efficient and robust tool for ATMT based gene deletion construction in fungi. Fungal Genet. Biol. 48, 677–684. 10.1016/j.fgb.2011.02.003. 21362493

[B31] Pearson M. N. Beever R. E. Boine B. Arthur K. (2009). Mycoviruses of filamentous fungi and their relevance to plant pathology. Mol. Plant Pathol. 10, 115–128. 10.1111/j.1364-3703.2008.00503.x 19161358 PMC6640375

[B32] Pouralibaba H. R. Pérez-de-Luque A. Rubiales D. (2017). Histopathology of the infection on resistant and susceptible lentil accessions by two contrasting pathotypes of *Fusarium oxysporum f*. sp. lentis. Eur. J. Plant Pathol. 148, 53–63. 10.1007/s10658-016-1068-6

[B33] Prieto P. Moore G. Shaw P. (2007). Fluorescence *in situ* hybridization on vibratome sections of plant tissues. Nat. Protoc. 2, 1831–1838. 10.1038/nprot.2007.265 17641652

[B34] Ramos B. Alves-Santos F. M. García-Sánchez M. A. Martin-Rodrigues N. Eslava A. P. Díaz-Mínguez J. M. (2007). The gene coding for a new transcription factor (ftf1) of *Fusarium oxysporum* is only expressed during infection of common bean. Fungal Genet. Biol. 44, 864–876. 10.1016/j.fgb.2007.03.003 17462924

[B35] Sarmiento-Villamil J. L. Prieto P. Klosterman S. J. García-Pedrajas M. D. (2018). Characterization of two homeodomain transcription factors with critical but distinct roles in virulence in the vascular pathogen *Verticillium dahliae*. Mol. Plant Pathol. 19, 986–1004. 10.1111/mpp.12584 28727279 PMC6638091

[B36] Sharzei A. Banihashemi Z. Afsharifar A. (2007). Detection and characterization of a double-stranded RNA mycovirus in *Fusarium oxysporum f*. sp. melonis. Iran J. Plant Path. 43, 9–26.

[B37] Upasani M. L. Gurjar G. S. Kadoo N. Y. Gupta V. S. (2016). Dynamics of colonization and expression of pathogenicity related genes in *Fusarium oxysporum f*. sp. ciceri during chickpea vascular wilt disease progression. PLoS ONE 11:e0156490. 10.1371/journal.pone.0156490. 27227745 PMC4882060

[B38] Valverde R. A. Nameth S. T. Jordan R. L. (1990). Analysis of double-stranded-RNA for plant-virus diagnosis. Plant Dis. 74, 255–258.

[B39] Xie J. Jiang D. (2014). New insights into mycoviruses and exploration for the biological control of crop fungal diseases. Annu. Rev. Phytopathol. 52, 45–68. 10.1146/annurev-phyto-102313-050222 25001452

[B40] Yu X. Li B. Fu Y. Jiang D. Ghabrial S. A. Li G.. (2010). A geminivirus-related DNA mycovirus that confers hypovirulence to a plant pathogenic fungus. Proc. Natl. Acad. Sci. U.S.A. 107, 8387–8392. 10.1073/pnas.0913535107 20404139 PMC2889581

[B41] Yu X. Li B. Fu Y. Xie J. Cheng J. Ghabrial S. A.. (2013). Extracellular transmission of a DNA mycovirus and its use as a natural fungicide. Proc. Natl. Acad. Sci. U.S.A. 110, 1452–1457. 10.1073/pnas.1213755110 23297222 PMC3557086

[B42] Zamora P. Martín A. B. Dueñas M. San Martín R. Diez J. J. (2015). *Cryphonectria parasitica* isolates of the same vegetative compatibility type display different rates of transfer of CHV1 hypovirus. Eur. J. Plant Pathol. 143, 767–777. 10.1007/s10658-015-0727-3

[B43] Zvirin T. Herman R. Brotman Y. Denisov Y. Belausov E. Freeman S. (2010). Differential colonization and defence responses of resistant and susceptible melon lines infected by *Fusarium oxysporum* race 1·2. Plant Pathol. 59, 576–585. 10.1111/j.1365-3059.2009.02225.x

