# Supplementary material for: Mycovirus Fusarium oxysporum f. sp. dianthi Virus 1 Decreases the Colonizing Efficiency of Its Fungal Host
Source: Front Cell Infect Microbiol. 2019 Mar 12;9:51. doi: 10.3389/fcimb.2019.00051 (PMC6422920; doi:10.3389/fcimb.2019.00051)
Supplement: Supplementary file 1 [file Table_1.docx]

| **SUPPLEMENTARY TABLE 1 \| Percentage of the standardized area under the disease progress curve (sAUDPC)**^(y)^ | | | | |
| --- | --- | --- | --- | --- |
| Isolates inoculated^(x)^ | Carnation cultivars | | | |
|  | Candela | | | Pink Bijou |
| *Fod* 77-wt | 0.84^a^ | | 0.99^a^ | |
| *Fod* 77-GFP | 0.87^a^ | 0.96^a^ | | |

^(y)^Means followed by the same letter are not significantly different according to Fisher’s

Least significant difference (LSD) (*P* ≤0.05).

^(x)^*Fod* 77-wt: wild type strain *Fod* 77.

*Fod* 77-GFP: *Fod* 77 strain expressing the GFP fluorescent protein.
