# Supplementary material for: Mycovirus Fusarium oxysporum f. sp. dianthi Virus 1 Decreases the Colonizing Efficiency of Its Fungal Host
Source: Front Cell Infect Microbiol. 2019 Mar 12;9:51. doi: 10.3389/fcimb.2019.00051 (PMC6422920; doi:10.3389/fcimb.2019.00051)
Supplement: Supplementary file 2 [file Data_Sheet_1.docx]

*
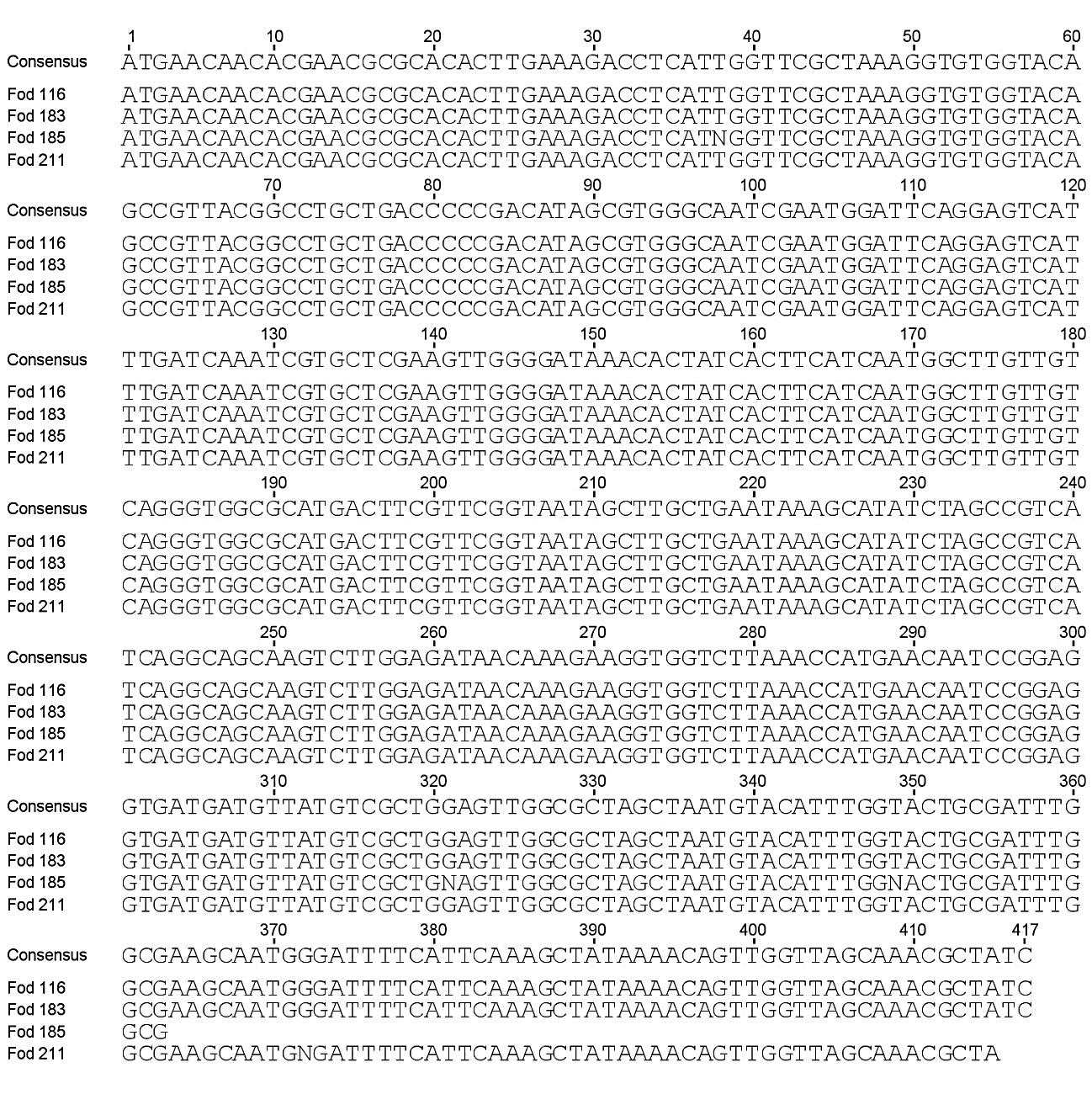
*

**SUPPLEMENTARY FIGURE 1 | Multiple alignment of the partial nucleotide sequence of the RdRp of FodV1.** Nucleotide sequence alignment of the amplicons obtained by RT-PCR using the dsRNA extracts from *Fusarium oxysporum* f. sp *dianthi* isolates *Fod* 116 (the strain originally infected with FodV1), *Fod* 183, *Fod* 185, and *Fod* 211. The alignment was carried out using the program Geneious version 8.1, created by Biomatters. The consensus sequence shows the identity between the amplicons used in the alignment.
