# Supplementary material for: Mycovirus Fusarium oxysporum f. sp. dianthi Virus 1 Decreases the Colonizing Efficiency of Its Fungal Host
Source: Front Cell Infect Microbiol. 2019 Mar 12;9:51. doi: 10.3389/fcimb.2019.00051 (PMC6422920; doi:10.3389/fcimb.2019.00051)
Supplement: Supplementary file 3 [file Data_Sheet_2.pdf]

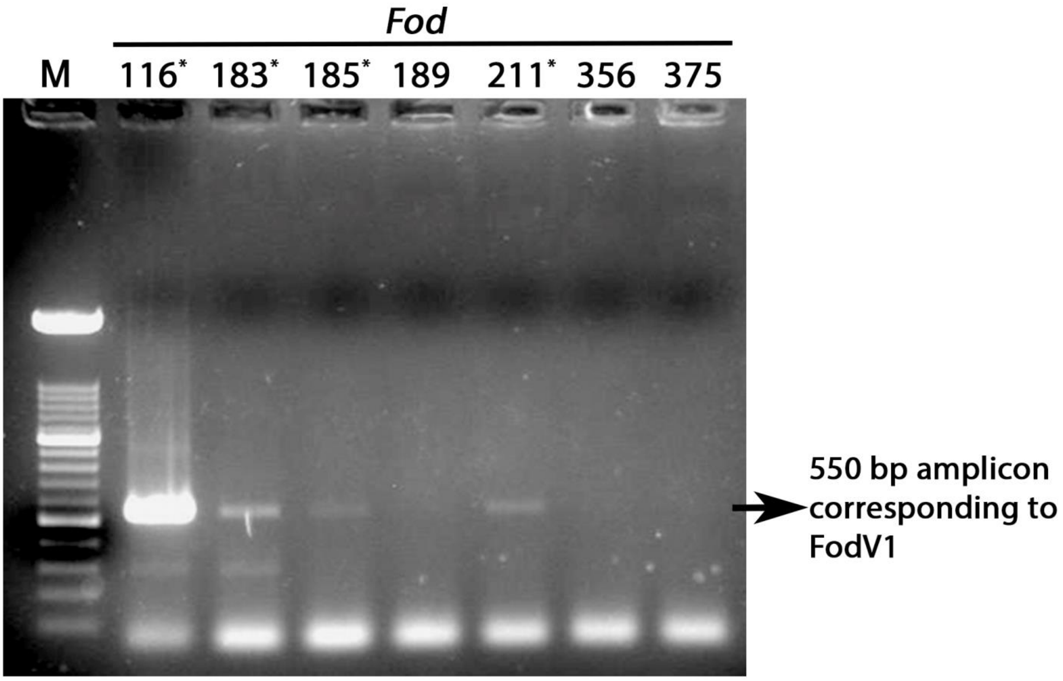

SUPPLEMENTARY IMAGE TO FIGURE 7 | Analysis of isolates of *Fusarium oxysporum* f. sp. *dianthi* (*Fod*) to identify infections with FodV1. Complete image corresponding to agarose gel electrophoresis of the RT-PCR products obtained using dsRNA extracts and specific primers for the RdRp segment of FodV1. * Isolates infected with mycovirus FodV1. **M**, molecular weight marker XIV (Roche Diagnostics).
